# How do BMI-restrictive policies impact women seeking NHS-funded IVF in the United Kingdom? A qualitative analysis of online forum discussions

**DOI:** 10.1186/s12978-024-01891-1

**Published:** 2024-10-28

**Authors:** Rebecca Muir, Meredith K. D. Hawking

**Affiliations:** Wolfson Institute of Population Health, Queen Mary University of London, London, UK

**Keywords:** Online forum analysis, In Vitro Fertilisation, Obesity, Healthcare access, Qualitative

## Abstract

**Background:**

Across the United Kingdom’s National Health Service (NHS), women with a Body Mass Index (BMI) of > 30 face restrictions accessing In Vitro Fertilisation (IVF) treatment. This study asks: what are the (un)expected and (un)intended harms and consequences experienced by women restricted from accessing NHS-funded IVF due to BMI threshold criteria?

**Methods:**

Posts from a popular infertility online forum were collected and reflexively thematically analysed.

**Results:**

On the forum, users discussed how they struggled to lose weight, how they faced time pressures to meet BMI thresholds, and they shared knowledge on how to comply or appear compliant with BMI cut-offs. Our study found widespread moral discourses around body weight were reproduced in the forum, particularly commonplace narratives that body weight is under personal control, that people with a high BMI should ‘work’ to change their bodies, and that this work helps demonstrate deservingness for IVF treatment. Moralising discourses around weight were linked to the responsibilities of a hoped-for future of motherhood, as users performed deservingness through emphasising their commitment to meeting the BMI threshold.

**Conclusion:**

We conclude that NHS-IVF policies in the United Kingdom do not consider the burdensome emotional and moral work placed on people seeking treatment due to inflexible upper-limit BMI criteria.

## Abstract (plain language summary)

In the United Kingdom, people can access public funding for In Vitro Fertilisation (IVF) treatment if certain criteria are met. Funding restrictions differ between geographical areas, but most areas restrict treatment to women with a Body Mass Index (BMI) below 30. This study explores the unexpected and unintended harms experienced by women restricted from NHS-funded IVF due to these BMI criteria. Posts from a popular infertility online forum were collected and thematically analysed. The study found moralising discourses around body weight which emphasized that women had personal control over their bodies and needed to ‘work’ to change their bodies to show deservingness for treatment. We conclude that NHS-IVF policies in the UK overlook the emotional and moral burdens placed on individuals due to rigid BMI criteria. As the impact of BMI limits on healthcare access is an under-researched topic, we believe this work is important for demonstrating the harms of BMI-restrictive policies.

## Introduction

Due to high patient demand and scarce NHS resources, NHS-funded In Vitro Fertilisation (IVF) treatment is rationed through restrictive access criteria such as BMI, age and ovarian reserve levels. While the NHS remains publicly committed to being a comprehensive service [1], restrictions on ‘low-priority’ treatments such as IVF have been criticised for representing a shift away from the notion of a universal and equally accessible NHS [2].

For several reasons, BMI restrictions on IVF access have been particularly under fire for the potentially far-reaching consequences of exclusion. The restriction of treatment based on BMI goes beyond what is recommended by national guidance (NICE, 2017). BMI limits on IVF access reflect a wider trend of BMI-based restrictions in NHS services [3]. The vast majority of local NHS commissioning bodies in the UK (discussed below) restrict IVF access to those with a BMI < 30 [4]. Approximately a quarter of women of reproductive age in the UK are categorised as having a BMI > 30 according to the NHS Health Survey for England [5].

BMI thresholds on IVF are present in various forms across western healthcare systems, and have been criticised for impacting marginalised communities disproportionately, including in Aotearoa New Zealand [6], and in the United States [7]. National data on how many people are turned away from NHS-IVF treatment due to not meeting BMI access criteria is not (to our knowledge) routinely collected. However, evidence points towards people with marginalised identities in the UK also being particularly impacted by access criteria. Studies of UK adult women have found that socio-economic deprivation and being of black ethnicity is statistically associated with higher BMI measurements [8, 9]. The HFEA report that NHS-funded IVF cycles decreased most among Black patients in 2021 and indicate this could be partially explained by locally implemented BMI restrictions [10]. It could be argued that BMI restrictions are a form of indirect discrimination on people with low financial means, as several private IVF providers in the UK allow treatment above BMI > 30.

Notable scientific and ethical analyses on BMI restrictions have concluded that BMI restrictions on access to IVF are not justified. Analyses by Brown [11] and separately by Tremellen and colleagues [12] has highlighted that the evidence-base does not provide a strong justification for excluding women with a high BMI on health and scientific grounds alone, and exclusion is not proportionate to the medical risk conferred by the treatment. It may be that obstetric risks, rather than IVF risks, are being considered in policymaking as several papers have found that high BMI may only weakly increase the risk of IVF complications [13–15]. The cost-effectiveness of BMI restrictions has also been scrutinised; as fertility problems can be highly distressing, people excluded from fertility care may require further support from NHS mental health and counselling services [16]. Additionally, recent clinical trials have found that weight-loss does not necessarily lead to better pregnancy outcomes for women labelled as obese or their children [17–19]. A review of pre-conception weight loss interventions amongst women classified as overweight or obese found that although natural conception rates may be positively affected by lifestyle and diet interventions, many people rapidly regain weight after an intervention ends [20]. Fluctuating weight regain is not ideal for patients undergoing multiple IVF cycles over a sustained period, particularly if they are subject to strict upper BMI thresholds limiting treatment access. These papers and conclusions cast doubt over whether recommendations for women to reduce their BMI before fertility treatment are medically justified.

Despite high levels of publicly visible activism and advocacy around the perceived unfairness of IVF restrictions, the experiences and perspectives of women restricted from accessing NHS IVF are currently under researched. In this paper, our aim was to examine how BMI-IVF restrictions are discussed and experienced, with a focus on (un)expected and (un)intended consequences and harms.

## Relevant national guidance on access criteria for IVF treatment

In the UK, the National Institute for Health and Care Excellence (NICE) provides clinical guidelines to aid in the diagnosis and treatment of fertility problems, and outline guidance around BMI (Box 1). Regional decision-making bodies known as Integrated Care Boards (ICBs) (previously Clinical Commissioning Groups (CCGs), interpret this guidance and manage the budget of NHS health services in the local area, which has led to the development of varying IVF funding policies across the UK.

Current NICE fertility guidelines on BMI (NICE, 2013, revised in 2017, Box 1)

**Table:** 

BMI-related fertility guidelines from: Fertility problems: assessment and treatment. Clinical guideline [CG156] Published: 20 February 2013 Last updated: 6 September 2017.
1.2.6.1 Women who have a body mass index (BMI) of 30 or over should be informed that they are likely to take longer to conceive. [2004, amended 2013]
1.2.6.2 Women who have a BMI of 30 or over and who are not ovulating should be informed that losing weight is likely to increase their chance of conception. [2004, amended 2013]
1.2.6.3 Women should be informed that participating in a group programme involving exercise and dietary advice leads to more pregnancies than weight loss advice alone. [2004]
1.10.4 Women should be informed that female BMI should ideally be in the range 19 to 30 before commencing assisted reproduction, and that a female BMI outside this range is likely to reduce the success of assisted reproduction procedures. [2004]

NICE claim to use ‘the best possible evidence’ to make judgements on clinical effectiveness, cost effectiveness and patient benefit to guide their decision-making around IVF access [21]. As IVF and related fertility technologies involve the potential creation of a new life, fertility care is recognised as difficult to assess with standard ‘cost effectiveness’ measures such as Quality-Adjusted Life Years (QALYs) [11], and thus capturing all the benefits and harms of treatment requires particularly careful, sensitive deliberation over what the costs, consequences and harms of IVF restrictions are for the people affected by BMI threshold policies. Although in no form of guidance does NICE explicitly recommend excluding women outside the BMI 19–30 range from IVF treatment, the vast majority of ICBs restrict women with a BMI outside the range of 19–30 from accessing NHS-funded IVF treatment [4], with some women excluded when their BMI is outside the range of 18–25 in some areas [22]. Additionally, local care pathways often direct potential patients with BMI > 30 to weight management services or interventions and can require women to undertake significant weight-loss before they can be re-referred to fertility services [23, 24].

BMI is not the only access criterion used to restrict IVF treatment in the NHS. For example, chronological age is also used to limit access. Age-related fertility guidance from NICE fertility guidelines states that women under 40 years are entitled to 3 full cycles of IVF, and that women aged 40 to 42 years should be offered 1 full cycle [25].

## Theoretical framework

It is being increasingly recognised in public health research that health policies can insufficiently assess or neglect to recognise potential harms experienced by people subjected to the policy first-hand [26–28]. Even well-intentioned health policies designed to reduce clinical risk or utilise public resources effectively can have unintended or counterintuitive effects. The evidence-based policy paradigm privileges certain types of knowledge in decision-making processes and can disembody knowledge from its social context [29]. As discussed by Cavanagh and Brehony, by becoming attuned to the ‘dark logic’ of a policy, policymakers and researchers can deepen their ethical engagement with research, and move beyond abstract discussions of “what works”, to a richer understanding of “what works, for whom, and under what circumstances” [30].

As critical social scientists, our analysis is particularly attuned to how the BMI policy may harmfully impact women in indirect ways, such as through cultural discourses reinforcing potentially harmful messages around body, weight, and health. We interpret harms through a social constructionist paradigm, which emphasises how concepts and categories are socially and historically situated and sustained through social processes [31]. Moral discourses can impact how healthcare policies are constructed, experienced and implemented. Moralising discourses around obesity have been identified in government obesity strategies [32], the UK press [33], and public health programmes [34]The shift towards neoliberal modes of healthcare governing has been criticised for creating a scarce NHS funding landscape, so that people who are perceived as failing to take care of their health are seen as unworthy of accessing stretched healthcare services [35, 36].

In this paper we also draw upon Hochschild’s theory of emotional work [37] to understand the impact of the BMI-restrictive policy. This theory suggests that the self is an emotional manager; individuals invest considerable effort to shape their inner emotions and to perform emotions appropriately to meet social expectations. Therefore, we also look at the socially performative elements in relation to adherence to the BMI policy.

## Methods

We analysed public forum posts on the topic of BMI criteria used to restrict IVF access in the UK NHS. Online forum analysis offers several advantages compared to other qualitative techniques such as interviews. Forums are a naturalistic environment where participants exchange information and reveal personal experiences. Weight is a sensitive topic for many women and in an interview, women might not feel comfortable disclosing their feelings about their bodies and weight-loss efforts. Additionally, unlike in a traditional research setting, discussions are not constrained or influenced by discourses produced by researcher interests. Forums hide identities and offer a level of anonymity to patients, which can encourage discussion of difficult or stigmatising topics. Additionally, forums are used to discuss clinical experiences in the ‘here and now’, as participants actively seek and contribute information, in contrast to interviews where often participants are expected to recollect and remember their health-related journeys [38].

In line with previous online forum research and guidelines [39] explicit informed consent was not required for this study as the forum was publicly accessible, did not need a login to access posts, and could be classed as ‘in the public domain’. Nevertheless, the respect and dignity of users was considered by maintaining privacy through robust procedures. In line with previous forum research, quotes were paraphrased so that they were less likely to be searchable via popular search engines [40] and this was checked by an independent researcher. This study obtained ethical approval from Queen Mary Ethics of Research Committee (QMERC22.185).

The forum was searched with the terms ‘BMI’ and ‘IVF’ to find relevant threads and 292 threads were found. For inclusion, threads must have been contributed to in the previous three years and include some discussion of the BMI > 30 access criteria in the original post with at least one responding relevant comment. Threads were given a time boundary between June 2019-June 2022. Selected threads were centred around the conceiving woman’s BMI (not the BMI of a male partner). Threads were excluded if they referred to the BMI threshold policy in regard to underweight exclusion (BMI < 18). After manually checking titles and posts for relevancy, a total of 217 individual posts were included in the study. Thread initial posts and replies constituted the units of analysis, rather than focussing specifically on an individual user’s contribution. This was because the forum included many transient users who might only post once and thus a log of their contributions would not have been possible. It was not possible to identify demographic characteristics of forum users as they are encouraged to not share personal information when signing up to post on the forum.

All threads were downloaded into word documents and deidentified through removal of usernames, pictures, names, and other information that could lead to personal identification. Usernames were replaced with forum post numbers for ease of analysis and kept in a separate password-protected document. Word documents were then numerically labelled and imported into NVivo 12 for analysis.

Information power was used to determine sample size [41]. Information power considers the broadness of the study aim, whether an established theory guides the research, the sample specificity, the quality of the dialogue, and strategy for analysis. Firstly, although the study specifically addresses women’s experiences of the BMI threshold when seeking IVF, we were not considering a specific demographic sample e.g., women with a low socioeconomic status. Therefore, we consider the aim to be broad, and considering a range of different life experiences. However, we were also restricted by the specificity of the topic. Due to the nature of online communication, the quality and richness of ‘dialogue’ present is likely to vary significantly from post to post. Due to this being an area of research not covered previously, the study was exploratory and focused on cross-case analysis to uncover varied experiences. We therefore aimed to include 30–50 threads, with the final sample including 33 threads.

Reflexive thematic analysis (RTA) was undertaken by RM to analyse forum posts, with MH acting as a ‘critical friend’ and discussant throughout the process. RTA emphasises the researcher’s active engagement to form overarching analytic themes [42]. This approach has six phases: becoming familiar with the data, code generation, theme construction, revising themes, and producing a cohesive interpretation of the data in a report.

Coding was both inductive, as we identified patterns in the data that addressed important issues represented in the text, and deductive, as the focus was on any (un)intended, (un)expected, and negative consequences of the BMI-restrictive policy. A combination of semantic and latent coding was used, to capture both harms described explicitly by forum users, and harms revealed through identifying underlying concepts, meanings and assumptions in the posts. Initial themes were created and then developed in reference to the existing literature.

## Results

**Table 1:** Summary of themes emerging from the online forum analysis

Themes				
1. Desperation to comply in time	2. Performing deservingness and taking responsibility	3. Feelings and emotional burdens	4. Knowledge seeking and sharing	5. Critical views of the BMI measurement

## Theme 1: desperation to comply in time

An overview of themes is available in Table 1. The first theme explores how BMI restrictions on access to IVF services enact power and control over women’s bodies by constricting time. Women expressed feeling the temporal pressure of declining biological fertility, and felt desperate to find ways to either meet or imitate compliance to the BMI criterion. Time was perceived as ‘running out’ by women drawing closer to being excluded by age restrictions on IVF access, and those who could not bypass the BMI policy to access private treatment. It was clear that having a BMI over 30 lengthened the time it took to access treatment, and this had significant consequences for women’s reproductive lives.

*‘I am at BMI of about 34 and I know weight loss takes a long time*,* there is also a significant waiting list*,* so I could be waiting 2–3 years before I could get started with treatment’ (FP38).*

There was a sense of urgency to lose weight to meet the BMI threshold. Some women called it the ‘race against time’ to lose weight and to have treatment sooner rather than later.

Although the age-based restriction criteria for NHS-funded IVF varies by geographical area, it was assumed knowledge in the forum that women older than 40 were not likely to be able to access NHS-funded IVF. There was also a general understanding that biological fertility declines with age, also affecting private treatment outcomes in those over 40 years old.

*‘I am in my early 40’s and have experienced unexplained infertility I do not have time to waste’ (FP78).*

*‘I am concerned about age-related complications*,* and feel that the trade-off between the risks of IVF at a higher weight are preferable to age-related risks’ (FP196).*

Our analysis shows how the age and BMI criteria collided to force women to manage a difficult balance of different types of ‘risk’, putting women under pressure to either make the choice that potentially maximises their chance at conceiving (if private treatment was a viable financial option), or to lose weight for the threshold quickly to meet the demands of the BMI policy. Time to wait for treatment was also extended by NHS waiting times. The below quote demonstrates how difficult this trade-off could be for some, as the BMI threshold in this example delayed treatment causing significant financial expense and regret for the forum user:

*‘I am 38 years old and had a BMI of mid-thirties when infertility was diagnosed. My doctor recommended private care instead of waiting if this was affordable. I “finished” losing the weight two months before my 40th birthday but could not access funding as I would not complete their treatment before I turned 40*,* a requirement of my local funding commissioner. This had significant financial consequences as I had to remortgage my home. I am active and healthy*,* just “big”’ (FP142).*

Overall, the threshold caused an additional layer of pressure on the limited time left to access IVF, and required women to weigh up their personal financial resources and any perceived medical risks from obesity against the threat of running out of time.

Users also demonstrated their compliance, or imitated compliance, through a variety of methods. This further demonstrated the desperation some users felt to comply with the BMI threshold. For instance, on the day of weighing at the clinic, forum users reported using different techniques to manipulate BMI measurements and meet the inclusion criteria. This included not eating or drinking before the weigh-in, limiting water for days before their appointment, sitting in the sauna and exercising before the appointment, wearing light clothes and standing up straight to extend height.

*‘I would compare the weight of my clothing to see which items were lightest’ (FP169).*

Others mentioned how healthcare professionals were complicit in manipulating measurements, whether it was taking the lowest reading or actively changing the result, indicating professionals make subjective judgements.

*‘I think discretion plays a large role as my doctor changed my BMI to 30 from 32.3’ (FP127).*

### Theme 2: performing deservingness and taking responsibility

The second theme explores how feelings of self-blame for being above the BMI threshold were evident in the forum. Women were keenly aware that NHS resources were scarce and enacted bodily practices to make sure their chance at conceiving were maximised. For instance, some women advised others against exercising as they suggested it could build heavy muscles, as this forum user explains:

*‘Combined with a low-calorie diet*,* I have completely stopped exercising to reach the BMI limit of 30*,* because muscle weighs more than fat and the BMI formula does not consider body composition’ (FP173).*

Others were clear that extreme weight-loss had negatively affected their fertility. Similarly, some users were also concerned that conditions such as polycystic ovarian syndrome (PCOS) would affect their weight-loss.

*‘My diet has been detrimental to my fertility because losing a significant amount of weight affected my hormones so they are “all out of whack”’ (FP68).*

Women discussed how ‘precious’ their chance to conceive felt to them and wanted to show others how important this opportunity was. Feelings of being undeserving of treatment were also present in the forum. They reflected how women internalised social and medical messages about weight and motherhood. The below example also demonstrates an experience of weight stigma, as the poster feels devalued due to the BMI categorisation process.

*‘I noticed that others in the forum have mentioned the BMI threshold*,* and I question what would happen if I could not meet the threshold. Am I not worthy to be a mum and is this journey a waste of time and hope?’ (FP160).*

In reply to threads centred around BMI worries, some women put forward the idea that healthcare professionals would identify more deserving candidates for IVF if effort was shown.

*‘Doctors can tell there is a genuine effort to meet the BMI requirements they might individually make a judgement call’ (FP151).*

Women also emphasised to others that they had a social responsibility to lose weight for the threshold due to the scarcity of NHS funding in reproductive care.

*‘Unless there is a good chance of treatment working*,* then the NHS would not want to use taxpayer money*,* I know you would want to give it your best possible try’ (FP136).*

Women also felt responsibility towards the health of their potential future child, and this was reinforced by peers on the forum. Women motivated each other by presenting an imagined future of a healthy child. This played on the duty women felt to provide an optimal environment for their potential baby. The work to get pregnant was likened to the work of raising a child, as losing weight becomes a facet of good motherhood, even before a baby is conceived.

*‘You have the best motivation going*,* and you should imagine that you are going through this experience to care for any future baby.’ (FP185)*.

*‘Diet plays an important part in fertility*,* so to maximise your chance and give our babies the best start in life (correct brain development) we need to lessen inflammation and maximise nutrition’ (FP58).*

This theme can be considered through the lens of ‘techniques of futuring’ (Oomen et al., 2017). From this perspective, a particular vision of the future is socially performed in the present and made socially authoritative through reinforcement. By imagining a new ‘storyline’ about the future, this can help orient others into action towards this future. Women posting on the forum outwardly hoped and worked towards a chance at a successful pregnancy. Users helped others keep engaged with weight-loss efforts by encouraging members of the forum to *‘focus on the present to take steps towards your dreams’* (FP188). This futuring can also be understood as a form of emotional work, as users reorient others into imagining an idealised future where weight-loss efforts have resulted in a healthy pregnancy and child.

## Theme 3: feelings and emotional burdens

The third theme focussed on the strong feelings of emotions expressed throughout the forum, as women used the online space to vent frustrations about the healthcare system and offer peer support. Some users expressed that they felt dismissed and judged by the healthcare system, leading to feelings of hopelessness.

*‘I have been bombarded with messages that I am too big and old and so I might as well stop putting in effort’ (FP160).*

In this theme, we found that BMI thresholds cause a significant amount of stress in women’s lives, amongst an already emotionally turbulent experience. Some felt it was the ‘*most stressful thing about IVF’ (FP1)* with one user writing that she blamed her weight for her previous IVF round failure, and was trying to internally challenge this guilt as they knew stress was not helpful for conceiving.

*‘I decided to be kinder to myself as I thought my unsuccessful previous round was due to my high BMI*,* and I was trying not to stress as I felt this would be detrimental to future attempts at conceiving’ (FP160).*

The above woman undertook ‘emotional work’ [37] ‘to be kinder to herself’ as she wanted to mitigate any negative psychosocial impact on her chance of pregnancy. Women also expressed contrition and regret for not meeting the BMI criteria. They ascribed previous failures to conceive as weight-related, and were critical of their past lifestyle and diet choices:

*‘It is my final chance to go through treatment with my own eggs and I don’t want to ruin it. Already I am beating myself up about treatment delays’ (FP165).*

One common source of worry was with regards to uncertainties in when weighing in clinical spaces might occur, and whether it occurred more than once. A few users wrote that they felt stuck, as they were not losing weight at the pace they needed for the criteria.

*‘I am trying so hard but weight loss efforts do not seem to be working*,* and I am worried. I am scared that I won’t be able to go ahead with my next IVF cycle’ (FP43).*

Emotional work complicated the experience of losing weight for the BMI threshold, for example in this case one woman advised another to stop worrying as this may affect the outcome of the IVF procedure:

*‘I am motivated to share my own experience so that you don’t get too stressed about weight*,* as worrying could affect your results’ (FP3).*

One woman spoke about the distress and feelings of dismissal after losing a very significant amount of weight but still failing to meet the criteria:

*‘I reduced my BMI down 9 BMI points and still was refused a referral. I felt I could not breathe when I heard this news’ (FP157)*.

Some women wrote about their difficult relationships with eating, and how stress from IVF and external life events exacerbated emotional eating. For many women, eating patterns had significant psychological dimensions. One user discussed undergoing therapeutic work to manage this issue, motivated by aiming to meet the criteria, demonstrating the additional types of patient work that emerged in the process. There were also unexpected mental health consequences to consider for some, as the process prompted some women to grapple with long-term disordered eating, or undertake an exploration of their relationship with food.

## Theme 4: knowledge seeking and sharing

This theme found that users sought more information about the BMI threshold on the forum, and shared their own knowledge. Women wanted to seek practical advice on losing weight and were also interested in finding out more about the rationale behind the policy, indicating this hadn’t been communicated thoroughly in healthcare settings. Women often used the forum to seek and share advice around how to lose weight. Advice ranged from generic weight-loss advice such as joining commercial slimming groups or trying to achieve a calorie deficit, to more fringe advice geared towards fertility such as going ‘chemical free’ or avoiding sugar.

*‘I am quite confused with what I am meant to eat*,* as on one hand I was told to relax and forget about it. Is dairy fine to eat or not? I’m lost’ (FP218).*

Users had questions about why BMI criteria were in place and a variety of explanations were offered. It was clear there was no straightforward or clearly understood rationale.

*‘I want more information about what the problem would be with excess weight- is it more difficult to produce eggs*,* or does it affect other aspects of conception?’ (FP32).*

Some users discussed how the lack of information from healthcare professionals confused them about risks to any potential pregnancy.

*‘If excess weight causes potential issues then surely the healthcare professionals are meant to mention this? I wonder if the lack of information on BMI means it is not that important.’ (FP196).*

*‘I spoke to my consultant and found out that losing excess weight would take pressure off ovaries*,* enhance egg production*,* and even when fallopian tubes are blocked*,* taking the excess tissue ‘out of the equation’ makes conception easier’ (FP74).*

Some users shared that private providers did not focus or often mention BMI or weight-loss.

*‘I was told by the NHS to lose weight*,* but the private clinic suggested that it really was not a great idea’ (FP68).*

*‘The private clinic was more interested in me having a good diet and did not weigh me throughout my time at the clinic. The clinic said IVF outcomes had nothing to do with BMI.’ (FP156).*

### Theme 5: critical views of the BMI measurement

Discussions around weight struggles were often accompanied by critical perspectives of the BMI as a tool used in medicine to understand and measure health and IVF success. A popular view was that BMI was not a good indicator of personal physical health. A variety of reasons were discussed, including belonging to a non-white ethnic group or having a broader body shape.

*‘I am not slim*,* and phone dieting apps have suggested that healthy weight loss would take 18 to 24 months. I would look unwell at the “healthy” BMI point and when I have reached a similar weight before I was told to gain weight by my GP’ (FP173).*

Others felt that the BMI threshold was arbitrary or motivated by wider financial scarcity issues with the NHS. Some also contrasted the NHS BMI criteria against more relaxed private healthcare criteria to highlight feelings of unfairness about the policy.

*‘The measure is “a load of crap” used to whittle down patients’ (FP156).*

Some voiced that the IVF process was ‘a constant list of do’s and don’ts’ and ‘endless rules’, with the BMI criteria being yet another barrier to receiving treatment. Others reluctantly accepted the criteria and saw it as simply hoops to jump through.

*‘I am confident that without the threshold*,* no health care professional would say dieting before IVF is good*,* but it was just the reality of what is needed to be done to meet threshold requirements’ (FP12).*

Another element of the discussion was how some felt BMI measures offered simple or easy explanations for infertility.

*‘My clinic had “no answer” for my struggles with conceiving but focussed on weight because the clinic said they were too busy to further investigate the issue’ (FP157).*

Some users resisted the negative messaging around obesity that they felt implicit within the BMI policy. These women rejected that weight was responsible for their fertility issues, and they advised others to take caution.

*‘I did not want to be weighed again as the process was demeaning and I felt “un-human”. I am not fat*,* I am beautiful*,* and infertility does not discriminate’ (FP196).*

### Discussion of harms

#### The harms of deservingness and the ‘good’ IVF patient

The bodily practices, including dieting work undertaken by women affected by the BMI access criteria for IVF is extensive. It encompasses: joining slimming groups; tracking calories on apps; increasing step-counts; and other diet and lifestyle-related practices that require high amounts of self-regulation and monitoring. The bodily practices also demonstrated effort and deservingness to others, as those who had lost significant amounts of weight were congratulated and reassured that they were maximising their chances of conceiving and giving a healthy start to a potential future child. Some users urged others to bring evidence of their weight-loss efforts to appointments, while others implied that healthcare professionals would see ‘effort’ put into reaching the criteria and react in positive ways towards this. The forum reflected common discourses around obesity, and particularly the generally accepted idea that body weight is under personal control, responsive to individual behaviour change, and that people in larger bodies should put in significant work to change their size. This focus on ‘trying anything possible’ to lose weight for treatment access reflects the broader IVF sociological literature, which emphasises how hope for a future baby or reproductive resolution remains pivotal in patients’ drive to pursue IVF [43, 44].

Findings also reflect studies on weight stigma, which examine how judgements of (un)deservingness are used to restrict people in larger bodies from medical treatment. For example, Wardell and colleagues found that people in larger bodies seeking funding for medical procedures through public crowdfunding positioned themselves as morally worthy for funding by emphasizing their efforts to ‘tame’ their body through exercise, dieting and by visually confessing their weight-related suffering, shame, and regret [45]. Similarly, Owen-Smith and colleagues [46] found that clinicians in a UK bariatric surgery clinic held a conviction that patients had to ’earn’ the right to surgery, while their patients gave ‘guilt-ridden, confessional’ narratives apologising for their size to convince gatekeepers of their deservingness.

Additionally, moralising discourses around what constitutes a ‘good mother’ were revealed in our analysis. Warin and colleagues researched Australian media sources and theorised that a new wave of ‘mother blame’ impacts reproductive care access, as women attempting to get pregnant are perceived as responsible for ‘passing on’ obesity to future generations [47]. Warin argues that the ‘mother blame’ discourse requires women to avoid this risk by creating optimal conditions for fertility through diet and nutrition. Consequently, women are held morally culpable for adverse health outcomes amongst their offspring, particularly if these women are labelled as overweight or obese and not seen to normatively regulate their bodies.

In our analysis, a fear of failure was evident throughout posts, and it appeared that users internalised blame for previous cycle failures and linked this to their difficulties in reaching the BMI access threshold. However, it is well-recognised that IVF has a low success rate – the HFEA states that around three quarters of IVF treatment is unsuccessful – and success is affected by a multitude of factors outside of personal control [48]. We suggest that these feelings of self-blame are another emotional consequence of the moral discourses around obesity and motherhood that we uncovered in the forum discussions. BMI-restrictive policies reproduce both weight stigmatising and ‘mother blame’ discourses, and this is an unexamined harm of the policies.

### Harms of rationing by delaying care

The BMI threshold led to users reporting delayed access to IVF services in a ‘race against time’. Private IVF treatments offer an alternative to these delays, as many clinics across the UK and Europe have more relaxed BMI criteria, although this varies according to clinic. Women in the forum analysis discussed the differences between NHS and private IVF related care, and many indicated that they were considering seeking private treatment or had accessed it due to more lenient clinic policies regarding weight. Delaying care also had the consequence of some women potentially facing age-related NHS IVF funding restrictions, therefore making them ineligible for publicly funded treatment.

It was not possible with our research to assess whether policymakers are aware that women may seek private treatment, and thus remove pressure on NHS resources if they are restricted from accessing IVF due to BMI, or if the real-world consequences of this implicit rationing mechanism are considered in IVF policymaking. Of course, the NHS and local commissioners must make difficult and complex decisions around funding allocation across multiple population and disease groups, and the explicit rationing practices already used in fertility care are controversial and often criticised by the media and the public. This could indicate that unpopular ‘explicit’ decisions around access are occasionally sidestepped through (un)acknowledged care delays.

Baldini and colleagues [49] investigated the risks of egg retrieval in higher weight patients, and found that oocyte retrieval rates in patients with BMI > 35 were not statistically different to the control group (BMI < 25). They suggest that to mitigate the impact of treatment delays due to high BMI, women could undergo egg retrieval at a higher weight followed by embryo cryopreservation, so that embryo transfer can occur later when weight loss is achieved. While this might be an achievable and realistic process for some women, we argue that delaying care through withholding the latter part of the treatment until weight-loss is achieved would not avoid the emotional and moral harms described in our work.

### Harms by targeting weight and displacing health

Policymakers may have intended for women to engage in healthier dieting practices and take up exercise regimes to achieve sustainable weight-loss and improve metabolic health, thus improving their chances of IVF success. However, in practice, due to biological fertility pressures, age-related IVF restrictions, and NHS waiting times, women may be incentivised to lose weight fast through extreme dieting and exercise practices.

Some women in the forum discussed their long-term weight struggles during their journey to accessing IVF. Wider literature notes the detrimental ‘yo-yo’ effect of dieting on health, as dieters who lose large amounts of weight and then gain back this weight again put stress on the cardiometabolic system and can increase risk for metabolic disorders such as diabetes [50, 51]. Low-calorie dieting has also been shown to increase long-term psychological stress and cortisol production, which is associated with weight-gain [52].

Some users in the forum appeared to struggle emotionally with the IVF access criteria. Contrary to the internal logic of some weight-loss interventions e.g [53], feelings of shame around weight do not necessarily motivate people to lose weight [54]. Weight stigma may partially explain why emotional eating patterns were evident and women found it so difficult to lose weight to achieve the BMI threshold. Experimental studies have found those experiencing weight stigma increase food intake and have decreased motivation to eat well [54]. The impact of weight-related shame is another un(intended) consequence of BMI thresholds used as a restrictive criterion.

This study demonstrates the power of online forum data for understanding people’s health experiences. Nevertheless, it is difficult to explore this topic fully with the online forum dataset due to varying depth of discussions available and the inability to elicit rich narratives through this retrospective approach. As we also could not confirm user’s identities, we recognise this is an additional limitation of our method. One consequence of the lack of identifying demographic data is that we were unable to draw conclusions about how racial marginalisation intersects with BMI thresholds and IVF access HFEA data shows that UK Black, Asian and Ethnic minority fertility patients start IVF treatment at older ages, access fewer NHS-funded IVF cycles, and have overall worse treatment outcomes, compared to their white counterparts [10]. Future studies should consider how people in the UK with marginalised racial identities are impacted by BMI restrictions. Research could also further explore how moral narratives around weight are constructed, circulated and reinforced by seekers of fertility care, peer-support networks, and by providers of fertility care services, through interviews or other deeper qualitative work.

## Conclusions

In practice, local commissioning bodies interpret NICE recommendations in such a way that it leads to complete exclusion of women with a BMI > 30 from NHS IVF treatment. Therefore, an (un)intended consequence of national recommendations is that IVF treatment access is dependent on weight-loss for women with a BMI > 30, often at an unsustainable pace due to other forms of restrictions, such as age-based criteria. Alongside other literature demonstrating weight-loss is unsustainable and potentially futile for many women’s IVF success, this research paper has shown the harmful consequences of BMI restrictions. Recommendations on IVF access which are presented as morally neutral do not consider the detrimental effects of weight-loss efforts on women’s emotional wellbeing and health. Furthermore, by hinging treatment access on a BMI threshold, women are forced into performing compliance and deservingness, and take on personal responsibility for their lack of access. BMI-related IVF restrictions create moral and emotional burdensome work for women, forcing them to position themselves as ‘good’ and ‘deserving’ potential IVF patients before they can begin accessing NHS funded care.

Research on patient perspectives in the United States has indicated that women restricted from IVF due to high BMI would like ‘actionable assistance’ from healthcare providers [55]. However, safe and equitable options are not currently available to women seeking support through the NHS. Current evidence suggests that pharmacological treatments, such as the GLP-1 agonist medicines (e.g. Semaglutide, Liraglutide, Tirzepatide), should not be offered to women in the preconception period as it may lead to increased risk of fetal abnormalities [56, 57]. The British National Formulary, used by clinicians in the NHS, states that the manufacturer of Semaglutide advises against pregnancy when taking the medication due to toxicity observed in animal studies [58]. While bariatric surgery has also been posited as a treatment option, a recent systematic review found there is limited good quality data around the impact of the surgery on live birth rates [59]. As the surgery results in a permanently altered digestive system, bariatric patients can face physical and psychological complications such as malnutrition, intense digestive system pain, and negative cognitions about the self, suggesting this could be a clinically inappropriate treatment for many women [60, 61]. Additionally, a recent investigation into NHS provision of weight loss services found that over a third of patients cannot get appointments with specialist teams for weight loss support, and seven out of 42 ICB’s do not fund bariatric surgery [62].

BMI restrictions in western healthcare systems appear to disproportionately impact minority groups and those with low socioeconomic status, and thus further reinforce long-standing health inequalities. Therefore, it is imperative that future policy reviews incorporate reproductive justice principles [7]. To aid this policy process, more empirical work needs to be done in the UK to understand how BMI, race, and socioeconomic status impact NHS-IVF access. Evidence reviewers should also synthesise diverse forms of evidence, including ethical analyses of BMI policies, sociological studies demonstrating the difficulties of weight-loss, and patient perspectives on BMI restrictions.

To our knowledge, there are no published studies investigating how BMI-based restrictive policies on IVF impact women in the United Kingdom. At the time of writing, NICE fertility guidelines are currently under review and have the potential to address weight-based restrictions. We suggest that rapid evidence gathering should take place. The guideline committee could undertake policy focus groups with people affected by BMI restrictions, or conduct a supplementary scoping literature review centred around the impact of weight discrimination in healthcare. Overall, our work shows that policymaking does not adequately consider the lived experiences of people subject to restrictions on NHS-IVF funding, and by failing to do so causes unjustified harm.

## Data Availability

No datasets were generated or analysed during the current study.

## Abbreviations

BMI: Body mass index
IVF: In Vitro Fertilisation
NHS: National Health Service
